# Complete chloroplast genome sequence of *Mirabilis himalaica* (Nyctaginaceae)

**DOI:** 10.1080/23802359.2019.1688116

**Published:** 2019-11-13

**Authors:** Shuli Wang, Chaonan Cai, Hui Ma, Jie Li

**Affiliations:** a Tibet Agricultural and Animal Husbandry University, Nyingchi, PR China;; b Center for Integrative Conservation, Plant Phylogenetics and Conservation Group, Xishuangbanna Tropical Botanical Garden, Chinese Academy of Sciences, Kunming, PR China;; c University of Chinese Academy of Sciences, Beijing, PR China

**Keywords:** *Mirabilis himalaica*, chloroplast genome, phylogenetic, *Nyctaginaceae*

## Abstract

*Mirabilis himalaica* (Nyctaginaceae) is endemic to the Himalayas where it is used in traditional Tibetan folk medicine. In this study, we first presented the complete chloroplast genome of *M. himalaica*. Complete genome size of *M. himalaica* ranged from 154,348 to 154,388 bp. The length varied from 85,808 to 85,845 bp in the (large single-copy) LSC region, from 17,935 to 17,938 bp in the (small single-copy) SSC region, and from 25,302 to 25,303 bp in the inverted repeat (IR) region. The overall GC contents of the chloroplast genome sequences were around 36%. Annotation analysis revealed a total of 112 genes, including 78 protein-coding genes, 30 tRNA genes, and 4 rRNA genes. The phylogenetic analysis with three *M. himalaica* samples and five other Nyctaginaceae species showed that *Mirabilis* including two species was clustered with high bootstrap support. The complete chloroplast genome sequences obtained in this study will provide valuable data for wider studies into the phylogenetics and conservation biology of *M. himalaica*.

*Mirabilis himalaica* (Edgew.) Heimerl belongs to the family Nyctaginaceae and is endemic to the Himalayas (Guan 1983; Lu and Gilbert 2003). Its roots are used as important Tibetan folk medicine for the treatment of nephritis edematous, renal calculus, arthrodynia, and uterine cancer (Yang 1991; Linghu et al. 2014). However, the wild populations are under pressure from market demand and serious human disturbance correspondingly. In order to conserve this valuable species, further study on the population genetics of *M. himalaica* is needed. Nevertheless, our understanding of the chloroplast genome of this species is limited. In this study, we first obtained the complete chloroplast genome of *M. himalaica* and explored the phylogenetic relationships with other Nyctaginaceae species, which will contribute to phylogenetics and conservation research of this taxon.

Three *M. himalaica* samples were collected from Xizang (Shannan and Linzhi) and Sichuan (Ganzi) province. All vouchers were deposited in the Research Institute of Xizang Plateau Ecology Herbarium (XZE) (Voucher No. 201501). Total DNA was extracted from dried leaves using the 3*CTAB method (Wang and Li 2007). High-quality DNA was used to build shotgun sequencing with Illumina Hiseq 2500 System at the Beijing Genomics Institute. After removed low-quality reads, high-quality reads were assembled by GetOrganelle (Jin et al. 2018). Gene annotation was conducted using an online Dual Organellar Genome Annotator (DOGMA) tool (Wyman et al. 2004). The *M. himalaica* chloroplast genome sequences were submitted to GenBank (number: MN548767–MN548769).

Chloroplast genomes displayed the typical quadripartite structure, including the small single-copy (SSC) region and large single-copy (LSC) region, separated by two inverted repeat (IR) regions (Palmer 1985; Daniell et al. 2016). The GC contents of the chloroplast genomes were relatively conserved and around 36%. Among those chloroplast genomes, genome size ranged from 154,348 to 154,388 bp. The length varied from 85,808 to 85,845 bp in the LSC region, from 17,935 to 17,938 bp in the SSC region, from 25,302 to 25,303 bp in the IR region. Each chloroplast genome contained 112 genes, including 78 protein-coding genes, 30 tRNA genes, and 4 rRNA genes. The protein-coding genes, rRNA genes, and tRNA genes accounted for 69.6%, 3.6%, and 26.8% of all annotated genes, respectively.

To study the phylogenetic relationships between *M. himalaica* and other species from Nyctaginaceae, five previously reported complete chloroplast genomes were downloaded in GenBank (Yao et al. 2019). A total of eight chloroplast genome sequences were aligned using MAFFT version 7.427 (Katoh and Standley 2013). The maximum likelihood (ML) analysis was performed using GTR model in RAxML version 8.2.10 (Stamatakis 2014), with 1000 bootstrap replicates to obtain support values. The phylogenetic tree showed that all the *M. himalaica* samples formed a terminal clade with strong support and two *Mirabilis* species containing *M. himalaica* and *Mirabilis jalapa* L. were also clustered with high bootstrap support (BS = 100, Figure 1). Although more comprehensive sampling across *Mirabilis* L is needed to resolve the phylogenetic relationships of the genus, the complete chloroplast genome of *M. himalaica* provides essential and important molecular database for further research on the phylogenetics and conservation biology.

**Figure 1.**
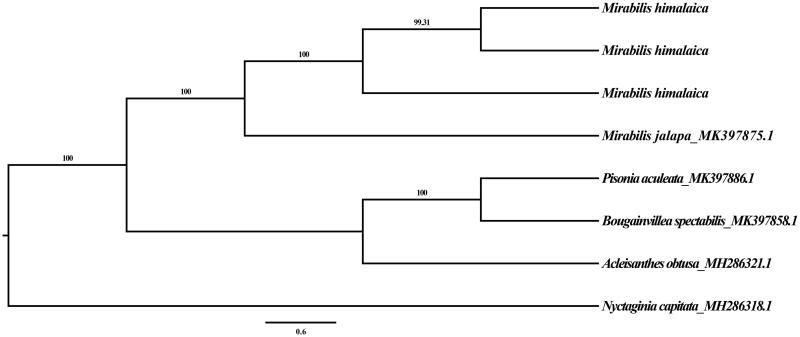
Maximum-likelihood (ML) topology from the complete chloroplast genomes of *M. himalaica* and the other 5 *Nyctaginaceae* species. Bootstrap support values greater than 50% are shown above the branches.
